# Evidence-Based Clinical Management of Canine Cognitive Dysfunction Syndrome: Diagnostic Algorithms, Practical Guidelines, Critical Appraisal of Biomarkers and Translational Limitations

**DOI:** 10.3390/ani16071114

**Published:** 2026-04-04

**Authors:** Maurizio Dondi, Ezio Bianchi, Paolo Borghetti, Valentina Buffagni, Rosanna Di Lecce, Giacomo Gnudi, Chiara Guarnieri, Francesca Ravanetti, Roberta Saleri, Attilio Corradi

**Affiliations:** Department of Veterinary Science, University of Parma, 43126 Parma, Italy; maurizio.dondi@unipr.it (M.D.); paolo.borghetti@unipr.it (P.B.); valentina.buffagni@unipr.it (V.B.); rosanna.dilecce@unipr.it (R.D.L.); giacomo.gnudi@unipr.it (G.G.); chiara.guarnieri1@unipr.it (C.G.); francesca.ravanetti@unipr.it (F.R.); roberta.saleri@unipr.it (R.S.); attilio.corradi@unipr.it (A.C.)

**Keywords:** cognitive dysfunction, dog, neurodegeneration, Alzheimer’s disease, amyloid-β, cerebral atrophy, DISHAA, CADES, neurofilament light chain, environmental enrichment

## Abstract

Canine Cognitive Dysfunction Syndrome (CCDS) is a progressive neurodegenerative disease affecting older dogs that shares many pathological mechanisms with human Alzheimer’s disease (AD). Although it is common in geriatric dogs, CCDS is often underdiagnosed in veterinary medicine. Both CCDS and AD involve a gradual decline in cognitive functions such as memory, learning and executive abilities. From a pathological perspective, dogs with CCDS show brain changes similar to those seen in AD, including cerebral atrophy, loss of neurons and accumulation of amyloid-beta plaques. CCDS is diagnosed by exclusion, meaning that other medical or neurological conditions that could cause similar behavioural signs must first be ruled out. Clinical evaluation mainly relies on structured questionnaires completed by owners. Magnetic resonance imaging is used to confirm cerebral atrophy and, at the same time, to exclude other brain disorders, such as cerebrovascular accidents and neoplasia. Current research focuses on identifying fluid biomarkers, such as amyloid-beta, neurofilament light chain and glial fibrillary acidic protein, to support an early and objective diagnosis. The most effective management combines pharmacological therapy, targeted nutrition and non-pharmacological strategies, including environmental enrichment and behavioural support. Early intervention, ideally during mild cognitive impairment, is crucial to slow disease progression and maintain quality of life.

## Simple Summary

Canine Cognitive Dysfunction Syndrome, often referred to as “canine dementia”, is a progressive neurodegenerative disease that affects older dogs. It manifests itself as a set of behavioural changes linked to a gradual decline in cognitive functions, which are not dependent on other medical or neurological diseases. It typically appears after 8 years of age, but the age of onset varies according to size: large dogs may show the first signs as early as 6–7 years, while small dogs more often show the first signs between 8 and 10 years. Laboratory studies have also found cognitive deficits, such as short-term memory problems, even in younger dogs, suggesting that changes begin before symptoms are apparent. Canine Cognitive Dysfunction Syndrome has a significant impact on a dog’s quality of life, causing confusion, anxiety and reduced interaction with the environment. It also negatively affects owners emotionally, practically and financially. Behaviours such as soiling indoors, night-time vocalisations and changes in social relationships can compromise the human–animal bond, influencing difficult decisions about long-term management. Therefore, recognising and managing Canine Cognitive Dysfunction Syndrome is essential for the well-being of the dog and the family.

## 1. Introduction

### 1.1. Definition and Clinical Significance of Canine Cognitive Dysfunction Syndrome (CCDS)

Canine Cognitive Dysfunction Syndrome (CCDS), often colloquially referred to as ‘canine dementia’, is a progressive neurodegenerative disorder that affects older dogs [1,2,3]. It is formally defined as the emergence of a set of behavioural signs indicating a gradual decline in cognitive function that cannot be attributed to other medical, neurological or behavioural conditions [4,5,6]. CCDS typically manifests in dogs aged eight years or older, although onset can vary by breed size. Large breeds may show signs as early as six to seven years of age, whereas small breeds are often considered to reach this threshold at eight to ten years [1,3]. Notably, laboratory studies have identified cognitive impairments, including short-term memory deficits, in Beagles as young as six years old, indicating that subclinical changes may precede the behavioural symptoms recognised by owners [5].

The clinical significance of CCDS extends far beyond the individual affected. It profoundly and negatively impacts the dog’s quality of life, leading to confusion, anxiety and diminished interaction with its environment [7,8]. It also imposes a significant emotional, practical and financial burden on owners and caregivers [1,7]. Behavioural changes associated with CCDS, such as house-soiling, nocturnal vocalisation and altered social interactions, can severely strain the human–animal bond, a cornerstone of the relationship with companion animals [5,9]. This erosion of the bond is a critical factor in veterinary medicine as it can influence owners’ decisions regarding long-term care and, in severe cases, may lead to relinquishment or premature euthanasia. Therefore, recognising and managing CCDS is crucial not only for animal welfare, but also for preserving the familial role of the ageing companion dog.

### 1.2. Epidemiology and Prevalence: An Underdiagnosed Geriatric Condition

CCDS is a highly prevalent condition within the geriatric canine population, yet it remains one of the most underdiagnosed diseases in veterinary practice. Epidemiological studies report a wide prevalence range, with the condition affecting between 14% and over 60% of dogs over eight years old [4,6,7,10]. The risk of developing the syndrome increases exponentially with age [4,10]. Data from specific cohorts illustrates this stark age-dependent increase; one study reported a prevalence of 28% in dogs aged 11–12 years, escalating to 68% in those aged 15–16 years [6,10]. A large-scale investigation as part of the Dog Aging Project further quantified this risk, finding that the odds of a dog having CCDS increased by 52% with each additional year of life [11].

Despite its high prevalence, the rate of formal clinical diagnosis is alarmingly low. A survey of almost 500 dogs aged eight to 19 years found that 14.2% had CCDS; however, only 1.9% of these cases had previously been diagnosed by a vet [10]. This significant discrepancy between epidemiological prevalence and clinical diagnosis is a major welfare issue in geriatric veterinary care. The primary reason for this ‘diagnostic gap’ is the common misconception among owners that subtle early behaviour changes in CCDS, such as mild confusion, increased sleep or slight withdrawal, are simply ‘normal’ or inevitable consequences of ageing [10,12]. This normalisation of pathological signs leads to them being overlooked when reporting to veterinarians. Furthermore, a survey of veterinary professionals revealed that, although awareness of the disease is high, the low diagnostic rates suggest that opportunities for intervention are often missed, even when potential signs are present.

The consequences of this diagnostic gap are profound. A significant proportion of elderly dogs are left to cope with the progressive challenges of a neurodegenerative disease without access to medical or environmental support. This unmanaged progression leads to a diminished quality of life for the animal and an increased caregiver burden for the owner [7]. Failure to diagnose and manage CCDS represents a critical failure in providing comprehensive geriatric care. This highlights the urgent need for a paradigm shift in veterinary practice, with cognitive screening becoming a routine and integral part of all senior and geriatric wellness examinations.

### 1.3. The Canine as a Spontaneous Model for Human Alzheimer’s Disease (AD)

The domestic dog is increasingly recognised as a powerful and highly relevant spontaneous animal model for human Alzheimer’s disease (AD) [7,10]. Unlike induced or transgenic rodent models, which often only recapitulate specific aspects of AD pathology, dogs naturally develop CCDS, a neurodegenerative condition that shares numerous striking parallels with human AD at clinical, neuropathological and molecular levels [2,12,13]. This makes dogs an invaluable tool for translational research, particularly for studying the complex interplay between genetic predisposition and environmental factors in the development of age-related dementia [10,13]. One drawback of the canine model is that identification of cognitive dysfunction relies on owners’ ability to recognise the signs, distinguish them from those associated with normal brain ageing, and consequently seek veterinary advice [1].

The key similarities that validate this model are numerous. Both CCDS and AD are clinically characterised by a progressive decline in cognitive domains, including memory, learning and executive function [5,10]. Pathologically, the brains of dogs with CCDS exhibit many of the same hallmarks as those with AD, most notably widespread cerebral atrophy, selective neuronal loss and extracellular deposition of amyloid-beta (Aβ) plaques [11,12,14]. Furthermore, dogs share the same environment as their human companions, exposing them to similar lifestyle variables, environmental toxins and nutritional influences that are suspected to be risk factors for AD [10]. This makes the canine model particularly well suited to investigating environmental risk factors and evaluating the efficacy of preventive and therapeutic strategies, such as nutritional interventions or pharmacological agents, with high potential for translation to human medicine [12,13,15]. Therefore, the study of CCDS and comparison with AD not only promises to improve the health and welfare of ageing dogs, but also has the potential to accelerate the development of effective treatments for one of humanity’s most devastating neurodegenerative diseases.

## 2. Pathophysiology and Neuropathology

### 2.1. The Role of Amyloid-β (Aβ) Deposition: Plaques and Cerebral Amyloid Angiopathy

The number of studies conducted on dogs with CCDS remains limited compared to those conducted on other animal models and humans. Nevertheless, our understanding of the pathophysiology and neuropathology of this disorder is gradually improving.

A defining feature of CCDS neuropathology is the progressive accumulation of the amyloid-beta (Aβ) peptide in the brain parenchyma and associated vasculature with age [4,10]. In dogs, aberrant amyloidogenic processing of amyloid precursor protein (APP) via BACE1 and γ-secretase leads to overproduction of aggregation-prone Aβ42, which accumulates primarily in prefrontal, hippocampal, and association cortices [16,17,18]. The Aβ42, a 42-amino acid peptide has an identical sequence in dogs and humans, which further strengthens the comparative value of the canine model [19]. This peptide has a high propensity to misfold and aggregate, forming soluble oligomers and protofibrils, which ultimately form large, insoluble amyloid plaques [10]. The topography and burden of cortical Aβ plaques correlate with learning and memory impairment and with “CCDS stage” in behavioural assessments [16,20,21]. Aβ deposits manifest in two main forms. The first is senile plaques, which are found in the extracellular space of the brain parenchyma. These can be categorised as diffuse plaques, which are amorphous and lack a dense core, or dense-core plaques, which are more compact and fibrillar [9,10].

The second is cerebral amyloid angiopathy (CAA), where Aβ accumulates in the walls of cerebral arteries, arterioles and capillaries [12,14]. As in human CAA, canine vascular Aβ is linked to vessel wall thickening, smooth-muscle loss, microbleeds, and hypoperfusion, generating an oxidative and inflammatory microenvironment that can promote tau phosphorylation and hinder perivascular and glymphatic clearance of Aβ and tau seeds [22,23,24]. These vascular and clearance failures likely contribute to the progression from “benign” Aβ ageing to clinically manifest CCDS. Both senile plaques and CAA are consistently observed in the brains of aged dogs and are considered to be hallmarks of CCDS pathology. In some, but not all, studies, the density and distribution of these Aβ deposits have been shown to correlate with the severity of the observed clinical cognitive impairment [9,15,19,25] (Table 1).

As in human AD, soluble Aβ oligomers appear to be the most synaptotoxic species, disrupting long-term potentiation, NMDA signalling, and calcium homeostasis [26,27], and increased soluble Aβ has been demonstrated in aged canine brains and plasma [16,28]. Furthermore, soluble Aβ oligomers derived from the brains of dogs with CCDS have been demonstrated to be neurotoxic in in vitro cell culture systems. This suggests that these smaller, non-plaque aggregates may be a key driver of the neuronal dysfunction and death observed in the disease [10]. Canine presenilins (PSEN1/2) and APP–γ-secretase processing is highly conserved relative to humans [29] and dogs naturally accumulate Aβ42-rich plaques and cerebral amyloid angiopathy (CAA) without experimental manipulation [16,30]. Although presenilin mutations like those driving familial AD in humans have not been defined in CCDS [31], the shared pathway and clinicopathologic correlations strongly support dogs as a spontaneous amyloid-based model.

### 2.2. Tau Pathology: A Point of Divergence from Human Alzheimer’s Disease

Although there are clear parallels in Aβ pathology between CCDS and human AD, a critical point of divergence lies in the presentation of tau pathology. A diagnosis of human AD requires the presence of two core pathologies: extracellular Aβ plaques and intracellular neurofibrillary tangles (NFTs) [10]. These NFTs are composed of hyperphosphorylated forms of the microtubule-associated protein tau. By contrast, mature, well-defined NFTs are generally considered absent or extremely rare in the brains of dogs with CCDS, even in very old animals with severe cognitive decline and a heavy burden of Aβ plaques [10,12,13] (Table 1).

This does not mean that the tau protein is unaffected in the ageing canine brain. More sensitive immunohistochemical studies have identified some phosphorylated tau epitopes, indicating that the initial steps of the pathological tau cascade do occur [7]. Aged dogs can show hyperphosphorylated tau in cortical and hippocampal neurons and, occasionally, small aggregates often associated with Aβ plaques [17,32,33]. However, classical, widespread neurofibrillary tangles and a Braak-type laminar progression are generally absent [31]. It has therefore been hypothesised that dogs may not live long enough for these early-stage tau alterations to progress to the formation of full-blown NFTs [10]. Alternatively, there may be intrinsic differences in canine tau protein or its associated kinases and phosphatases that render it less prone to aggregation. Regardless of the underlying reason, this relative lack of advanced tauopathy is the most significant neuropathological difference between CCDS and classic human AD. This distinction has important implications for the use of the canine model in research. While the dog is an excellent model for studying Aβ-related pathogenesis and therapies, it may be less suitable for investigating therapeutic strategies that specifically target advanced tau aggregation and NFT formation [10].

### 2.3. Mechanisms: Oxidative Stress, Mitochondrial Dysfunction, and Neuroinflammation

Beyond structural changes such as plaque formation and atrophy, the pathophysiology of CCDS is driven by a cascade of harmful molecular events at the cellular level. A key element of this cascade is oxidative stress. Due to its high metabolic rate and lipid-rich composition, the brain is particularly susceptible to damage from reactive oxygen species (ROS) [5]. In the ageing canine brain, there is a progressive imbalance between ROS production and the capacity of endogenous antioxidant systems to neutralise them. This leads to cumulative oxidative damage to critical cellular components, including lipids, proteins and nucleic acids [4,12]. This heightened oxidative environment is closely linked to mitochondrial dysfunction. Mitochondria, the cell’s primary energy producers, are also a major source of endogenous ROS. In dogs with CCDS, neuronal mitochondria exhibit age-related defects, including increased ROS production and impaired bioenergetic function [7,34]. This results in a state of neuronal energy deficit, which is exacerbated by impaired glucose metabolism in the brain—a phenomenon also observed in human AD [7,25]. This bioenergetic failure compromises the ability of neurons to maintain ionic gradients, transmit signals and perform essential housekeeping functions, making them more susceptible to degeneration and death.

A third critical mechanism is neuroinflammation, which is now widely recognised as an active driver of the disease process, rather than merely a consequence of neurodegeneration. The presence of Aβ deposits and products of oxidative damage triggers a chronic inflammatory response triggered and amplified by the glial cells that work as brain’s resident “immune” cells: microglia (“first responders”) and astrocytes (“amplifiers and regulators”) [7,35]. In CCDS, these glial cells become activated and adopt a pro-inflammatory phenotype. The crosstalk between microglia and astrocytes, mainly via the release of pro-inflammatory cytokines production (IL-1, TNF-a, IL-6 and chemokines) determines whether neuroinflammation is protective or detrimental. While this is initially a protective response, sustained glial activation becomes maladaptive and contributes to neuronal damage by releasing inflammatory cytokines, chemokines and further ROS. Importantly, studies have demonstrated that the extent of microglial and astrocytic activation is more closely associated with the clinical severity of CCDS than the presence of Aβ plaques. This suggests that this inflammatory response may be a more direct cause of the observed cognitive impairments [9]. This finding challenges the traditional amyloid-centric view of pathogenesis and highlights neuroinflammation as a key therapeutic target.

More recently, the structure of the CNS glymphatic system and its role in the clearance of extracellular proteins have suggested that its dysfunction may play an important role in the pathogenesis of AD [36].

### 2.4. Alterations in Neurotransmitter Systems

The cognitive and behavioural deficits that define CCDS are the clinical manifestation of widespread synaptic dysfunction and imbalances in key neurotransmitter systems. These neurodegenerative processes do not occur in isolation; they directly affect the synthesis, release and reception of the chemical messengers essential for normal brain function.

One of the most well-documented alterations is a cholinergic deficit, which is a key finding in AD [2,10]. Evidence of degeneration and loss of cholinergic neurons originating in the basal forebrain, which project widely throughout the cortex and hippocampus, has been found [10]. This results in reduced levels of the neurotransmitter acetylcholine, which plays a critical role in learning, memory, and attention (Table 1). This cholinergic hypofunction provides the rationale for therapies aimed at boosting acetylcholine levels, such as cholinesterase inhibitors [2].

Other monoaminergic systems are also profoundly affected. The noradrenergic system, which plays a role in arousal, attention, and mood, undergoes degeneration [10]. Concurrently, there is an age-related increase in the activity of the monoamine oxidase B (MAO-B) enzyme [4]. This enzyme degrades catecholamines, particularly dopamine. Increased MAO-B activity leads to lower dopamine levels, which can contribute to apathy and altered motor activity. It also generates hydrogen peroxide as a by-product, which further contributes to oxidative stress [37]. This decline in dopamine levels is observed not only in CCDS and AD, but also in other neurodegenerative disorders in humans, particularly Parkinson’s disease (PD) [38]. This shared abnormality constitutes the basis for the use of MAO-B inhibitors such as selegiline in the treatment of CCDS as well as PD.

Furthermore, broader alterations in other systems, including GABAergic and serotonergic pathways, likely contribute to the complex behavioural phenotype of CCDS, particularly signs of anxiety and altered sleep–wake cycles [4].

### 2.5. Hallmarks of Neurodegeneration in the Canine Brain: Cerebral Atrophy and Neuronal Loss

The macroscopic neuropathological changes observed in the brains of dogs with CCDS are strikingly similar to those found in humans with AD. Post-mortem examinations and advanced imaging studies consistently reveal evidence of gross cerebral atrophy, characterised by narrowing of the cerebral gyri and widening of the sulci [2,12,39]. This loss of brain volume is often accompanied by compensatory enlargement of the ventricular system, a finding also common in human AD [2].

At microscopic level, this atrophy is the result of significant and irreversible neuronal loss. Histopathological analyses have identified the selective degeneration and death of neurons in key brain regions associated with higher cognitive functions [7]. The areas most profoundly affected include the cerebral cortex, particularly the prefrontal, parietal and occipital lobes, and the hippocampus, which is critical for memory formation and spatial navigation [10]. This pattern of neuronal loss directly correlates with the clinical signs observed in CCDS, such as disorientation, memory impairment and altered social behaviour (Table 1). Studies have demonstrated that apoptosis, or programmed cell death, significantly contributes to neuronal depletion in the ageing canine brain [25]. Histologically, diffuse Aβ plaques predominate, with cored/neuritic plaques more abundant in clinically affected dogs [16,30]. Neuronal loss and synaptic depletion, especially in frontal cortex and hippocampus, parallel cognitive decline [31,40], and white-matter rarefaction and demyelination are frequent in frontal regions [20]. Immunohistochemistry using human Aβ antibodies (4G8, 6E10, anti-Aβ42/40) robustly labels canine plaques and CAA, while synaptophysin and NeuN highlight synaptic and neuronal loss, and GFAP/Iba1 document plaque-associated astro- and microgliosis [16,32].

**Table 1:** CCDS neuropathology basis and neuroclinical relevance.

Feature	Neuropathology	Neuroclinical Relevance	References
Amyloid-β (Aβ) Deposition	Diffuse and cored Aβ plaques predominantly in prefrontal cortex, hippocampus, and association cortices; mainly Aβ42	Correlates with cognitive decline, spatial learning deficits, and executive dysfunction; parallels human AD topography	[16,17,18,30]
Soluble Aβ Oligomers	Increased soluble Aβ species in aged canine brains and plasma	Principal mediators of synaptic toxicity; disrupt LTP, NMDA signalling, and calcium homeostasis	[16,26,27,28]
Presenilin and APP Processing	Highly conserved canine PSEN1/2 and APP–γ-secretase pathway; spontaneous Aβ42 accumulation	Natural amyloidogenic processing without genetic manipulation; no yet defined presenilin mutations in CCDS as in AD	[16,29,31]
Tau Pathology	Hyperphosphorylated tau in cortex and hippocampus; scattered aggregates near Aβ plaques; rare classical NFTs	Limited, non-stereotyped tauopathy compared to human AD; CCDS is predominantly Aβ-centric	[17,31,32,33]
Cerebral Amyloid Angiopathy (CAA)	Aβ40 deposition in leptomeningeal and cortical vessel walls; common in aged dogs, more severe in CCDS	Vessel wall damage, microbleeds, hypoperfusion; impairs perivascular/glymphatic clearance	[14,24,30]
Brain Atrophy	Generalised cortical atrophy, especially frontal and temporal lobes; hippocampal volume loss; ventricular enlargement	Correlates with spatial learning and memory deficits; less severe than advanced human AD	[31,39]
Neuronal and Synaptic Loss	Neuronal loss and synaptic depletion in frontal cortex and hippocampus; reduced synaptophysin	Best structural correlate of cognitive impairment; parallels human AD pattern	[31,39]
White Matter Changes	Frontal white matter rarefaction and demyelination	Contributes to cognitive dysfunction; may reflect vascular and inflammatory processes	[20,39]
Cholinergic Dysfunction	Degeneration of basal forebrain cholinergic neurons; reduced choline acetyltransferase (ChAT) activity	Impaired learning, disorientation, sleep–wake disruption; therapeutic target for cholinesterase inhibitors	[2,10,27]

## 3. Clinical Manifestations and Diagnosis

### 3.1. The DISHAA Acronym: A Framework for Clinical Signs

The clinical presentation of CCDS is characterised by a diverse array of behavioural changes that reflect the widespread nature of the underlying neurodegeneration. To provide a structured framework for veterinarians and owners to recognise and categorise these signs, the acronym DISHAA has been widely adopted [7,10]. Each letter represents a core behavioural domain that is commonly affected in dogs with the syndrome.

**Figure 1:**
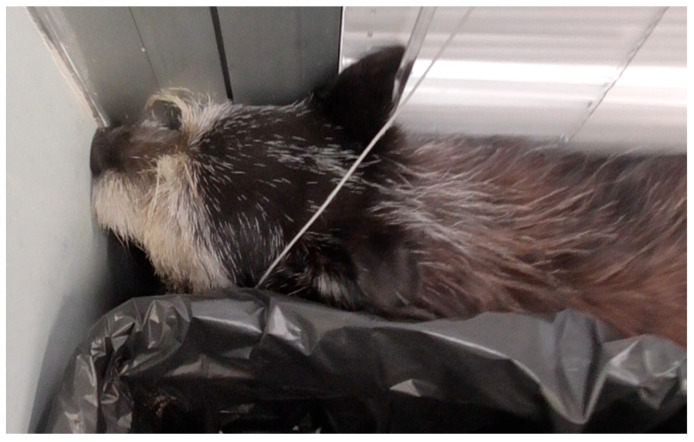
Image of an elderly dog with cognitive dysfunction, stuck in a corner of the room and unable to find a way out.

- D—Disorientation: This is one of the most classic signs of CCDS. Dogs may appear lost or confused in familiar environments, such as their own home or garden [4,10]. Common manifestations include staring blankly at walls or into space, becoming ‘stuck’ in corners or behind furniture and being unable to find a way out (Figure 1), and failing to respond to their name or familiar commands [9,12].
- I—Interactions (altered): The dog’s social behaviour towards owners, other pets and familiar people often changes significantly. This can manifest as decreased greeting behaviours, reduced desire for petting or social contact, or appearing more withdrawn [4,9,10]. Conversely, some dogs may become overly needy or ‘clingy’, showing an increase in attention-seeking behaviours. Irritability or uncharacteristic aggression can also emerge.
- S—Sleep–wake cycle (altered): Disruption of the normal circadian rhythm is a hallmark of the syndrome [7,9]. Affected dogs often sleep more during the day and experience periods of restlessness, pacing or vocalisation at night [4]. This nocturnal activity can be particularly distressing for owners and is a frequent reason for seeking veterinary consultation.
- H—House-soiling: This involves the dog urinating or defecating indoors, often in inappropriate or unusual locations, after previously being reliably housetrained [7,12]. This is not due to a lack of opportunity to eliminate outside, but rather a cognitive failure to recognise the appropriate location or signal the need to go out.
- A—Activity (altered): Changes in overall activity levels are common and can go in either direction. Many dogs exhibit a decrease in purposeful activity, showing apathy and reduced interest in exploration, play or walks [7,10]. Others may exhibit repetitive, aimless behaviours, such as constant pacing, wandering in circles or walking back and forth along a fixed path [41].
- A—Anxiety (increased): Many dogs with CCDS exhibit new or exacerbated signs of anxiety [7,10]. This may manifest as an increased fear of stimuli that were previously tolerated, the development of new phobias (e.g., thunderstorms or loud noises), or a heightened state of generalised anxiety [4,13]. Separation anxiety, in particular, may emerge or intensify, causing significant distress for both the dog and owner when left alone [13]. This anxiety component is a critical aspect of the clinical picture as it significantly impacts the animal’s welfare [4].

### 3.2. The Diagnostic Process: A Diagnosis of Exclusion

Currently, there is no definitive antemortem test that can confirm a diagnosis of CCDS. These diagnostic difficulties arise from a combination of the nature of this disorder and the limited amount of data available on advanced diagnostic methods that could distinguish CCDS from other possible diagnoses [12,15,42,43]. Consequently, diagnosis remains by exclusion, requiring a systematic and thorough approach to rule out other medical conditions that can cause or contribute to similar behavioural signs [3,4,13]. The absence of a standardised, universally accepted diagnostic protocol poses a significant challenge in clinical practice and is widely recognised as a need within the veterinary community [3]. The diagnostic process begins with taking a comprehensive medical history and conducting a complete physical and neurological examination. The veterinarian must meticulously investigate potential non-cognitive causes of the reported behavioural changes [10]. Furthermore, environmental factors may contribute to the development of primary behavioural disorders, resulting in symptoms also reported in CCDS, such as irritability, house soiling and anxiety [44].

Concerning systemic and neurological diseases that can mimic the signs of CCDS, key differential diagnoses include:

- Endocrine disorders: Hypothyroidism can cause lethargy and mental dullness, while hyperadrenocorticism (Cushing’s disease) can lead to house-soiling due to polyuria and nocturnal restlessness [10].
- Sensory decline: Progressive vision loss (e.g., due to cataracts or progressive retinal atrophy) or deafness may result in disorientation, increased anxiety and altered social interactions [10,44].
- Chronic pain: Undermanaged pain, most commonly from osteoarthritis, is a major confounding factor. A dog in pain may be reluctant to move, appear apathetic, become irritable when touched and be restless at night. They may also soil the house due to difficulty posturing or navigating stairs to get outside [13,44].
- Metabolic or organ-system disease: Conditions such as chronic kidney disease or hepatic encephalopathy can cause lethargy, confusion and other neurological signs.
- Intracranial disease: Structural lesions in the brain, particularly neoplasms (e.g., meningioma or glioma), are a critical differential diagnosis, especially in cases with a more acute onset or the presence of focal neurological deficits [10].

In order to effectively rule out these conditions, it is essential to obtain a minimum database including a complete blood count (CBC), serum chemistry profile, urinalysis and thyroid hormone levels [10,12]. Blood pressure measurement is also crucial, as systemic hypertension can have neurological consequences. If the initial workup yields no results but clinical suspicion remains high, further diagnostics such as advanced brain imaging (MRI) may be necessary to rule out structural lesions.

### 3.3. Validated Behavioural Assessment Tools: CCDR and CADES

Due to the behavioural nature of the diagnosis, the foundation of the clinical assessment of CCDS lies in the use of structured questionnaires or rating scales completed by owners [4,12]. These tools are designed to systematically quantify the presence, frequency and severity of the behavioural signs associated with the syndrome, providing a more objective scoring system than anecdotal owner reports. Several such instruments have been developed, but two have emerged as the most widely used and scientifically validated in the literature: the Canine Cognitive Dysfunction Rating (CCDR) scale and the CAnine DEmentia Scale (CADES) [45,46] (Table 2).

The CCDR scale is a well-established tool that assesses signs across several behavioural categories, such as disorientation, social interaction, and house-soiling [12,23,25]. It uses a scoring system to grade the frequency of abnormal behaviours, yielding a total score that can be used to classify a dog as cognitively normal, at risk/mildly impaired, or having CCDS [4,44,45].

CADES is a more recent tool developed to further refine the assessment process [2,46]. It evaluates behaviours across four distinct domains: spatial orientation, social interactions, sleep–wake cycles and house-soiling. Evidence suggests that the CADES may be superior to the CCDR for certain applications, particularly for staging disease severity, identifying earlier, more subtle stages of cognitive impairment and tracking clinical sign progression more sensitively over time [10].

Although these rating scales are invaluable clinical tools, they are limited by their reliance on owner observation and interpretation [12,45]. This introduces subjectivity, which can be influenced by the owner’s awareness, relationship with the dog and personal interpretation of what constitutes ‘normal’ ageing. Nevertheless, these scales currently represent the most practical and validated method for the presumptive antemortem diagnosis and monitoring of CCDS in a clinical setting.

### 3.4. Advanced Diagnostic Modalities

#### 3.4.1. Structural Magnetic Resonance Imaging (MRI): From Atrophy to Hippocampal Volumetrics

Advanced neuroimaging, particularly magnetic resonance imaging (MRI), plays a dual role in diagnosing CCDS in dogs. Its primary function is to rule out other structural brain diseases that can produce similar clinical signs, such as intracranial neoplasia, inflammatory brain disease or cerebrovascular accidents [10,44].

**Figure 2:**
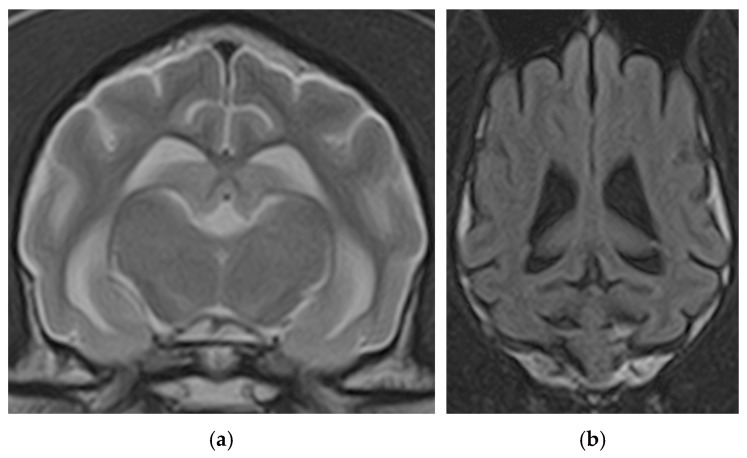
(**a**) Transverse T2-weighted and (**b**) dorsal fluid-attenuated inversion recovery (FLAIR) MRI of the brain of a dog with suspect CCDS showing widening of the cerebral sulci and enlargement of the lateral ventricles (courtesy of Dr. M. Bernardini, AniCura Veterinary Hospital “I Portoni Rossi”—Zola Predosa (BO) Italy).

Beyond exclusion, MRI can also provide evidence to support a diagnosis of CCDS. Qualitative assessment of MRI scans from affected dogs often reveals findings consistent with the known gross pathology of the disease, including generalised, age-inappropriate cerebral atrophy—visible as widening of the cerebral sulci and enlargement of the lateral ventricles [12,14]. While these findings are not specific to CCDS and can be seen in normal ageing, they may be more severe in cognitively impaired individuals (Figure 2).

More recently, quantitative MRI techniques have provided a more objective structural correlate for the disease. Specifically, volumetric analysis has demonstrated that dogs diagnosed with CCDS have a statistically significant reduction in total hippocampal volume compared to age-matched control dogs that are cognitively normal [42]. This finding of hippocampal atrophy directly parallels a key imaging biomarker used in the diagnosis of human AD and provides a structural basis for the memory deficits that are a core feature of CCDS [42]. However, it is important to note that there is a substantial degree of overlap in hippocampal volumes between the CCDS and control groups. This means that this measurement cannot currently be used to definitively diagnose or exclude CCDS on an individual patient basis [42]. Nevertheless, this represents a significant step towards identifying objective, imaging-based biomarkers for the disease.

#### 3.4.2. The Quest for Objective Biomarkers: CSF and Plasma Analytes (Aβ, NfL, GFAP)

Current CCDS research primarily focuses on identifying and validating fluid-based biomarkers that can provide an objective biological basis for diagnosis. This could potentially allow for earlier and more accurate detection than is possible using behavioural scales alone [3,4,12]. This research largely mirrors efforts in the field of human AD, focusing on analytes in cerebrospinal fluid (CSF) and blood (plasma or serum) that reflect the core underlying pathologies of the disease [12].

Amyloid-β (Aβ): Its central role in CCDS pathology makes Aβ a primary target for biomarkers. In line with the ‘sequestration’ hypothesis of AD, numerous studies have discovered that, as Aβ aggregates into plaques within the brain, its concentration in the cerebrospinal fluid (CSF) and plasma decreases [10,25]. Consequently, lower levels of Aβ42 and a reduced Aβ42/Aβ40 ratio in CSF or plasma are considered potential indicators of cerebral amyloidosis [4,15]. However, results from canine studies have been somewhat inconsistent, and the usefulness of plasma Aβ as a standalone diagnostic marker is still being investigated.

Neurofilament light chain (NfL): NfL is a structural protein of the neuronal axon. When neurons are damaged or die, NfL is released into the CSF and subsequently leaks into the peripheral circulation. It is therefore considered a sensitive, albeit non-specific, marker of neuroaxonal injury [15]. Recent studies have shown that CSF and plasma NfL concentrations are both significantly elevated in dogs with CCDS, and that these levels correlate positively with the severity of cognitive decline, as measured by behavioural questionnaires [15,43]. However, NfL’s lack of specificity is a key limitation, as elevated levels can also be observed in other neurological conditions, such as epilepsy or meningoencephalitis [15].

Glial Fibrillary Acidic Protein (GFAP): GFAP is an intermediate filament protein primarily found in astrocytes. The presence of GFAP in the CSF and blood indicates astrocytic activation (astrogliosis), which is a key component of the neuroinflammatory response in CCDS [15]. Consequently, elevated GFAP levels are being investigated as a potential biomarker of neuroinflammation. Studies have shown an increasing trend in plasma and CSF GFAP levels in dogs with CCDS, which supports its potential use as part of a biomarker panel [15,43].

Novel biomarkers: The search for more specific and sensitive biomarkers is ongoing. Recent research has explored various novel blood-based analytes, such as retinol-binding protein 4 (RBP4), C-X-C motif chemokine ligand 10 (CXCL10) and NADPH oxidase 4 (NOX4) [12]. Preliminary studies using machine learning algorithms suggest that combining measurements of several of these novel markers could significantly improve the accuracy with which CCDS can be diagnosed from a simple blood sample. The ultimate goal is to develop a panel of blood-based biomarkers that, when used alongside clinical assessments and behavioural scales, can provide an accessible and early definitive diagnosis of CCDS.

## 4. Therapeutic and Management Strategies

### 4.1. Pharmacological Interventions

#### 4.1.1. Selegiline Hydrochloride: A Monoamine Oxidase-B Inhibitor

Selegiline hydrochloride, also known as L-deprenyl, is an irreversible inhibitor of the enzyme monoamine oxidase B (MAO-B). It is currently the only pharmaceutical agent approved by the United States’ Food and Drug Administration (FDA) for treating the clinical signs associated with CCDS [37]. Its mechanism of action is multifaceted. Firstly, by inhibiting MAO-B, selegiline reduces the degradation of catecholamines, particularly dopamine, in the central nervous system. This enhances dopaminergic and other catecholaminergic neurotransmission in cortical and subcortical regions, potentially improving cognitive function and mood [37]. Additionally, selegiline is believed to exert neuroprotective effects. The catabolism of monoamines by MAO-B produces hydrogen peroxide and other reactive oxygen species. By inhibiting this process, selegiline reduces the production of oxidative free radicals and thus mitigates a key driver of neurodegeneration [37] (Table 3).

The clinical efficacy of selegiline has been demonstrated in several open-label field studies. In one large study involving 641 dogs with clinical signs of CCDS, 77.2% of cases showed an overall improvement after 60 days of treatment with selegiline [37]. Improvements were observed across multiple DISHAA categories, with the most significant benefits seen in terms of disorientation and altered interactions with family members. Clinical improvement is often noted by owners within the first 30 to 60 days of initiating therapy. Selegiline is generally well tolerated by geriatric patients. The most commonly reported adverse effects are typically mild and transient, and include gastrointestinal disturbances such as vomiting, diarrhoea or loss of appetite, as well as potential central nervous system effects such as restlessness or increased salivation [37].

It is not surprising that this MAO-B inhibitor is also widely used to treat human PD. Although CCDS differs from human PD in a number of clinical and neuropathological respects (such as the absence of Lewy bodies), various features are shared by human neurodegenerative disorders, which makes them part of a pathological continuum that ranges from AD to PD, encompassing other disorders such as Lewy body Dementia and Progressive supranuclear palsy [38,47].

#### 4.1.2. Propentofylline: A Xanthine Derivative with Neuroprotective Properties

Propentofylline, a xanthine derivative, is licenced in Europe and other regions for treating dullness, lethargy and other clinical signs of ageing in dogs [5,37]. Its mechanism of action is complex and targets several pathophysiological processes relevant to CCDS. It acts as a phosphodiesterase inhibitor, blocking the cellular uptake of adenosine and leading to vasodilation and improved cerebral blood flow [48]. This may help counteract age-related reductions in cerebral perfusion. Propentofylline also has rheological effects, inhibiting platelet aggregation and thus further enhancing microcirculation [5].

Beyond its vascular effects, propentofylline exhibits direct neuroprotective and anti-inflammatory properties. It has been shown to inhibit the production of free radicals and, crucially, to reduce the activation of microglial cells [5,48]. By modulating this key aspect of the neuroinflammatory cascade, propentofylline may help slow the progression of the underlying disease process rather than providing purely symptomatic relief [48] (Table 3). Although it is widely used by veterinarians and reported to improve general demeanour, lethargy and dullness in older dogs, the evidence base for its efficacy in improving the core cognitive domains of CCDS is limited. Some comparative studies have found it to be less effective than other agents at improving specific behavioural measures such as locomotion and exploratory activity in aged dogs [49].

#### 4.1.3. Emerging and Investigational Pharmacotherapies

The search for more effective treatments for CCDS is an active area of research, with several novel therapeutic strategies currently being investigated in preclinical and clinical trials. These strategies target different aspects of the disease’s complex pathophysiology.

- Cholinesterase inhibitors: Given the well-established cholinergic deficit in CCDS, increasing acetylcholine levels through therapy is a logical approach [2,10]. Although these inhibitors are a mainstay of symptomatic treatment for human AD, they have been used less frequently in veterinary medicine. However, a recent preliminary study investigating the use of a novel, selective butyrylcholinesterase (BChE) inhibitor in dogs with moderate CCDS demonstrated significant improvements in owner-assessed cognitive scores (CADES) and in performance on objective cognitive tests [2] (Table 3). These promising results suggest that targeting the cholinergic system may be a viable therapeutic strategy for CCDS.
- The TRAC Clinical Trial: A significant, large-scale clinical trial is currently underway at Colorado State University to evaluate three different investigational drugs for the treatment of CCDS [49]. This placebo-controlled trial is investigating the effects of three drugs: trazodone (a serotonin antagonist and reuptake inhibitor), rapamycin (an mTOR inhibitor with potential anti-ageing effects) and cannabidiol (CBD), which has purported anti-inflammatory and neuroprotective properties. This trial represents a major effort to systematically evaluate novel therapies that target distinct pathways implicated in brain ageing and neurodegeneration [50].
- Senolytics and NAD+ precursors: This novel therapeutic strategy involves targeting cellular senescence, which is the process whereby aged cells stop dividing and enter a pro-inflammatory state. A recent randomised, double-blind, placebo-controlled clinical trial evaluated the effectiveness of a combination product containing a senolytic agent to promote the clearance of senescent cells and a nicotinamide adenine dinucleotide (NAD+) precursor to support cellular energy metabolism [51] (Table 3). The study found that dogs in the full-dose treatment group showed a statistically significant improvement in owner-assessed cognitive function, as measured by a reduction in their CCDR scores over a three-month period compared with the placebo group [51].
- Transcranial photobiomodulation (tPBMT): Non-pharmacological interventions are also being explored, such as transcranial photobiomodulation therapy (tPBMT), which involves applying low-level light energy to the head. It is thought to improve mitochondrial function, reduce oxidative stress and decrease inflammation within the brain. A small prospective case series involving five dogs with moderate to severe CCDS evaluated the effects of a 60-day tPBMT protocol [8]. The results were promising: all five dogs showed improvement in their cognitive scores by the end of the study, with no adverse effects reported [8]. Further controlled studies are needed to validate these findings.

Although preliminary data on these emerging therapeutic strategies are encouraging, there is still little scientific evidence of their effectiveness in treating or slowing the progression of CCDS. Published reports on these therapies have important limitations, such as small sample sizes and a lack of long-term follow-up. Well-designed, large-scale, randomised, placebo-controlled trials are needed to support the evidence-based use of these drugs in CCDS.

### 4.2. Nutritional Management

#### 4.2.1. Therapeutic Diets and Key Nutraceuticals (MCTs, Omega-3 Fatty Acids, Antioxidants)

Nutritional intervention has emerged as a cornerstone of the multimodal management of CCDS, with a growing body of evidence supporting the use of specific dietary modifications and nutraceuticals to promote brain health and enhance cognitive function in ageing dogs [7]. These interventions are designed to counteract several key molecular mechanisms underlying CCDS, including impaired energy metabolism, oxidative stress and inflammation.

- Medium-chain triglycerides (MCTs): One of the most significant advances in the nutritional management of CCDS is the use of MCT-enriched diets [52]. The ageing brain in both dogs and humans exhibits impaired glucose metabolism, effectively starving neurons of their primary energy source. MCTs are readily metabolised by the liver into ketone bodies, which can cross the blood–brain barrier and serve as an alternative, readily available fuel source for the brain [52]. Clinical studies have demonstrated that feeding aged dogs diets containing 5.5% to 9% MCTs significantly improves cognitive performance, particularly in the areas of executive function, memory and learning [7,12,52] (Table 3).
- Omega-3 fatty acids: They are critical components of neuronal cell membranes and have potent anti-inflammatory properties (Table 3). Supplementing the diet with these fatty acids has been shown to improve cognitive function in ageing dogs, and evidence suggests that higher doses are more effective, particularly in enhancing learning ability [7].
- Antioxidants and mitochondrial cofactors: To combat the pervasive oxidative stress that characterises ageing, therapeutic diets for CCDS often contain a variety of antioxidants [15]. These typically include vitamins E and C, selenium and antioxidants derived from fruits and vegetables, such as flavonoids and carotenoids. Such diets may also contain mitochondrial cofactors such as L-carnitine and alpha-lipoic acid to support mitochondrial function and improve cellular energy production [34]. Clinical trials have demonstrated that the long-term administration of antioxidant-enriched diets can enhance learning and memory, as well as mitigating the behavioural manifestations of CCDS [15] (Table 3). While individual antioxidants such as vitamins E and C may be less effective on their own, they are essential for preventing the oxidative degradation of omega-3 fatty acids in the diet when included in a complex blend [7].
- Tramiprosate (Homotaurine): This small aminosulfonate compound binds to soluble Aβ, resulting in reduced amyloid aggregation and deposition. It also exhibits antioxidant, anti-inflammatory and neuroprotective properties in vivo and/or in vitro [53,54]. Dietary supplementation with tramiprosate, alongside the stimulation and exercise of a learning activity, appears to slow down cognitive ageing in dogs [53].
- Other promising nutraceuticals: Research has also identified other specific supplements that may be beneficial. S-adenosylmethionine (SAMe), a molecule involved in neurotransmitter synthesis and antioxidant pathways, has been shown in clinical trials to alleviate the signs of CCDS [5,7]. Apoaequorin, a calcium-binding protein originally isolated from jellyfish, has also been shown to have a positive effect on learning and executive function in elderly dogs [7]. The chronic administration of citicoline has been shown to facilitate learning and memory processes in dogs [5].

#### 4.2.2. Evidence Base for Nutritional Interventions

The evidence supporting the nutritional management of CCDS comes from a variety of studies, including laboratory-based trials and clinical studies involving client-owned dogs. A systematic review, published in 2025 and analysing 27 canine clinical trials conducted between 2002 and 2023, provides valuable insight into this body of research [7]. The review concluded that studies evaluating complete, enriched therapeutic diets generally demonstrated higher methodological quality than studies investigating single-ingredient supplements [7]. This suggests that a multimodal nutritional approach, in which multiple beneficial nutrients are combined into a single diet, may be more effective and is supported by more robust evidence. Although results from individual supplement studies can be inconsistent, collective evidence strongly supports integrating targeted nutritional strategies, particularly diets enriched with MCTs, antioxidants and omega-3 fatty acids, into the standard of care for dogs with or at risk of developing CCDS [7,14,52].

### 4.3. Non-Pharmacological Management

#### 4.3.1. Environmental Enrichment and Behavioural Modification

Non-pharmacological strategies that focus on a dog’s environment and daily activities are a critical—yet often overlooked—component of a comprehensive management plan for CCDS. Based on the principles of neuroplasticity and the ‘use it or lose it’ concept of brain health, these interventions aim to stimulate the brain and slow the progression of cognitive decline.

Environmental enrichment is a multifaceted strategy that increases the complexity and engagement of a dog’s daily life. It encompasses three key areas:

1. Cognitive enrichment: This involves providing mental stimulation through activities such as puzzle toys, food-dispensing toys, scent work and learning new simple commands or tricks [19,34].
2. Social enrichment: Ensuring regular positive social interaction with owners, and if appropriate, other familiar people and dogs [19].
3. Physical enrichment: This includes regular physical exercise, which has direct benefits, as well as exposure to novel environments and sensory stimuli. Examples include walks in different locations with varied sights, sounds and smells [34].

The benefits of environmental enrichment are not just theoretical. Laboratory-based studies in ageing Beagles have provided compelling evidence of its effectiveness. Long-term enrichment has been shown to significantly improve performance in cognitive tasks and preserve the number of neurons in the hippocampus, which is a key memory centre [19,34]. Furthermore, a landmark study demonstrated a powerful synergistic effect when environmental enrichment was combined with an antioxidant-fortified diet. This combination therapy was found to increase the expression of brain-derived neurotrophic factor (BDNF) mRNA in the brains of aged dogs to levels approaching those seen in young, healthy animals [19]. BDNF is a critical neurotrophin that supports the survival of existing neurons and encourages the growth and differentiation of new neurons and synapses. This makes it essential for learning, memory and overall brain health (Table 3).

Behavioural modification techniques are employed to manage specific problem behaviours and reduce anxiety. This involves establishing and maintaining predictable daily routines for feeding, walking and sleeping, which can reduce confusion and anxiety in cognitively impaired dogs (Table 3). Clear and consistent communication must also be re-established. This may require adapting training cues to accommodate sensory decline; for example, hand signals could be used for a hearing-impaired dog, and tactile cues for a visually impaired one. Positive reinforcement techniques using high-value rewards should be employed to encourage desirable behaviours and help the dog relearn lost commands or routines such as house training [5]. The ultimate aim is to create a safe, predictable and supportive environment that minimises stress and maximises the dog’s ability to function.

#### 4.3.2. The Role of Physical Activity

Physical activity warrants special mention as a standalone intervention due to the strength of the evidence supporting its role in promoting healthy ageing of the brain (Table 3). Large-scale observational studies have demonstrated a strong and significant correlation between a dog’s level of physical activity and cognitive health [11,13]. This is not merely a correlation; the data suggest a protective effect.

For example, a study from the Dog Aging Project, which included data from over 15,000 companion dogs, found that, after controlling for factors such as age, breed and health status, dogs reported by their owners as inactive were 6.47 times more likely to be diagnosed with CCDS than those reported as very active [11]. Another study found that higher levels of physical activity were not only associated with a lower prevalence of CCDS, but also with lower current symptom severity and a slower rate of symptom worsening over a six-month period [13]. The mechanisms underlying this benefit are likely to be multifactorial and include improved cerebral blood flow, reduced inflammation, the promotion of neurogenesis and increased expression of neurotrophic factors such as BDNF. While the physical limitations of geriatric dogs must be considered, maintaining an appropriate level of regular exercise is an effective, evidence-based strategy for reducing the risk and progression of CCDS.

### 4.4. A Multimodal Approach to Management

The complex and multifaceted nature of CCDS pathophysiology requires a multimodal therapeutic approach. The consensus derived from the scientific literature is that no single intervention is likely to be sufficient for optimal management. The most effective strategy is therefore to implement pharmacological treatments, targeted nutritional support and non-pharmacological interventions, such as environmental enrichment and behavioural management, concurrently [7,8,52]. This integrated approach allows multiple pathways involved in the disease process to be targeted simultaneously. For instance, a veterinarian may prescribe selegiline to address neurotransmitter imbalances, recommend a therapeutic diet enriched with medium-chain triglycerides (MCTs) and antioxidants to provide alternative energy sources and combat oxidative stress, and advise the owner to implement a programme of regular exercise and cognitive enrichment to stimulate neuroplasticity and increase brain-derived neurotrophic factor (BDNF) levels.

Early intervention is a critical element for the success of any management plan. The neurodegenerative changes in CCDS are progressive and mostly irreversible. Therefore, therapeutic measures should be instituted as early as possible in the disease course, ideally at the stage of mild cognitive impairment, to have the greatest positive clinical effect, slowing the rate of decline and preserving a good quality of life for a longer period [1,8]. This once again highlights the importance of proactive screening for cognitive decline in all senior dogs.

**Table 3:** Summary of Therapeutic Interventions for CCDS.

Intervention Category	Specific Intervention	Proposed Mechanism of Action	Summary of Evidence/Key Clinical Findings	References
Pharmacological	Selegiline Hydrochloride 0.5–1 mg/kg PO SID	Irreversible MAO-B inhibitor; increases dopamine/catecholamine levels; reduces oxidative stress.	Licenced in the US. Open-label trials show ~77% of dogs improve, particularly in disorientation and social interaction.	[5,37]
	Propentofylline 5 mg/kg PO BID	Xanthine derivative; improves cerebral blood flow; neuroprotective and anti-inflammatory via microglial modulation.	Licenced in Europe. Reported to improve dullness and lethargy; evidence for specific cognitive benefits is limited.	[5,48,49]
	Cholinesterase Inhibitors	Increase acetylcholine levels by inhibiting its breakdown, addressing the cholinergic deficit.	Investigational. A novel BChE inhibitor showed significant cognitive improvement in a preliminary canine study.	[2]
	Senolytics/NAD+ Precursors	Clear senescent cells and support cellular energy metabolism.	Investigational. A combination product significantly improved owner-assessed cognitive scores in an RCT.	[51]
Nutritional	MCT-Enriched Diet	Provides ketone bodies as an alternative energy source for the brain, bypassing impaired glucose metabolism.	Strong evidence from multiple clinical trials shows improvement in executive function, memory, and learning.	[7,12,14,52]
	Omega-3 Fatty Acids (DHA/EPA)	Structural component of neurons; potent anti-inflammatory effects.	Systematic review supports cognitive benefits, especially at higher doses, for learning functions.	[7]
	Antioxidant Blend	Neutralises reactive oxygen species, reducing oxidative damage to brain cells.	Diets fortified with a blend of antioxidants (Vitamins E and C, etc.) improve cognition and reduce brain pathology.	[15,34]
	Tramiprosate (Homotaurine)	Reduces amyloid aggregation and deposition. Antioxidant, anti-inflammatory and neuroprotective properties	Dietary supplementation with tramiprosate, alongside the stimulation and exercise of a learning activity, slow down cognitive ageing in dogs	[53,54]
Non-Pharmacological	Environmental Enrichment	Provides cognitive, social, and physical stimulation to promote neuroplasticity.	Improves cognitive performance; preserves hippocampal neurons. Synergistic with diet to increase BDNF levels.	[19,34]
	Physical Activity	Improves cerebral blood flow; increases neurotrophic factors; reduces inflammation	Strong association with better cognitive outcomes. Inactive dogs have >6× higher odds of CCDS.	[11,13]
	Behavioural Modification	Establishes predictable routines and uses positive reinforcement to reduce anxiety and manage problem behaviours.	Recommended as part of a multimodal plan to reduce stress and improve coping in cognitively impaired dogs.	[5]

## 5. Prognosis and Disease Progression

### 5.1. The Natural History of CCDS: From Mild Cognitive Impairment to Severe Dysfunction

By its nature, Canine Cognitive Dysfunction Syndrome is a progressive disorder [10,41]. The clinical course reflects the slow but inexorable progression of the underlying neurodegenerative pathology. Longitudinal studies that have observed cohorts of ageing dogs over several years have provided valuable insight into this progression. The disease does not usually present suddenly in its severe form, but rather follows a trajectory that often begins with a prodromal stage frequently referred to as mild cognitive impairment (MCI) [4].

During this MCI or ‘at-risk’ stage, dogs exhibit subtle behavioural changes that do not yet significantly impact their overall function, but which nonetheless represent a deviation from their previous norm. These early signs can be detected using validated rating scales such as the CADES or CCDR [4,41]. As the disease progresses, these mild signs become more frequent and severe, eventually crossing the threshold for a diagnosis of overt CCDS. A two-year longitudinal study clearly documented this natural progression: over the study period, 33% of dogs that were cognitively normal at the outset progressed to MCI, while 22% of dogs that began the study with MCI progressed to a CCDS diagnosis [15,41]. While this progression is generally the rule, it is important to recognise that the rate of cognitive decline can be highly variable among individual dogs. Some appear to remain stable for long periods, while others decline more rapidly [9,41].

### 5.2. Prognostic Indicators and Risk Factors

While the exact cause of CCDS is not fully understood, research has identified several factors associated with an increased risk of developing the disease, or which may influence its prognosis.

- Age is undoubtedly the most significant and consistently identified risk factor for CCDS [10,11]. The prevalence, incidence and severity of the disease all increase dramatically as dogs move into their senior and geriatric years.
- Physical activity: As previously discussed, physical inactivity is a major modifiable risk factor. A sedentary lifestyle is strongly and independently associated with a much higher likelihood of a CCDS diagnosis [11,13].
- Comorbidities: The presence of other health conditions, particularly those affecting the nervous system or sensory organs, is a significant risk factor. Dogs with a history of other neurological diseases, such as idiopathic epilepsy, or significant sensory impairment (e.g., blindness or deafness), are more likely to develop CCDS [10,11]. Furthermore, physical disturbances commonly found in older dogs, such as gait abnormalities, tremors or a decline in sense of smell, are also significantly associated with a CCDS diagnosis and may represent an early clinical indicator of the underlying neurodegenerative process [46].
- Nutrition: Diet appears to play a role in modulating risk. One study found that dogs fed a controlled, high-quality diet were significantly less likely to develop CCDS than those fed an uncontrolled, lower-quality diet [10].
- Other factors: The influence of demographic factors such as sex, reproductive status (neutered or not) and breed size has been investigated, but the results have been inconsistent across studies. Most studies have found no statistically significant association [6,10].

The strong association between CCDS and other age-related health problems challenges a simplistic view of the disease. The standard diagnostic approach is to exclude other medical conditions that could mimic the signs of CCDS. However, the data clearly show that these conditions are not merely mimics, but often true comorbidities [11,13]. For example, a dog with painful osteoarthritis may be less interactive and more restless at night; however, this pain can also act as a chronic stressor that may exacerbate underlying cognitive decline. Conversely, a cognitively impaired dog may be less able to cope with or communicate its pain. This interconnectedness suggests that CCDS should not be viewed as an isolated brain disease, but rather as a central component of a broader geriatric ‘frailty’ syndrome, in which multiple physiological systems decline concurrently. This perspective has significant clinical implications, shifting the focus from a single-disease diagnosis to a comprehensive geriatric assessment. Treating a dog’s arthritis or sensory deficits is not merely addressing a comorbidity; it is directly intervening to improve the dog’s overall state. This can positively impact its cognitive function by reducing the systemic ‘noise’ of pain and distress, and enhancing its ability to engage in enriching activities.

### 5.3. Impact on Quality of Life and Survival

It is beyond doubt that an advanced CCDS diagnosis significantly impacts the quality of life for affected dogs, leading to chronic confusion, anxiety and distress [7,8]. The condition also has a profound effect on the owner, creating a substantial care burden that can be emotionally and physically taxing.

Given the progressive and debilitating nature of the disease, one might expect it to be associated with a shortened lifespan. However, several observational studies have yielded a surprising finding: a CCDS diagnosis does not appear to reduce survival time [4,41]. In these longitudinal cohorts, the median survival time of dogs with CCDS was not statistically different to that of their cognitively normal, age-matched counterparts [3]. There are several potential explanations for this. Firstly, the disease typically has a late onset, meaning that many dogs may succumb to other age-related conditions before cognitive decline becomes life-limiting. Secondly, and perhaps more importantly, the strong human–animal bond may encourage owners of dogs with CCDS to provide exceptionally high levels of supportive care, which could positively influence longevity [3]. These findings suggest that, with the right veterinary care and owner support, many dogs with CCDS can enjoy a full natural lifespan.

## 6. Future Directions

### 6.1. Gaps in Current Knowledge and Areas for Future Research

Despite significant progress in understanding CCDS over the past two decades, several critical knowledge gaps remain, highlighting key areas for future research. One of the most urgent needs is the development and implementation of standardised, evidence-based and universally accepted clinical guidelines for diagnosing, staging and managing the syndrome [3]. These guidelines would help to close the ‘diagnostic gap’ and ensure a consistent standard of care for elderly patients.

A second major research imperative is the continued development, validation and refinement of objective, accessible and reliable biomarkers [12]. Although promising candidates such as plasma NfL, GFAP and Aβ ratios have been identified, further validation is required in large, diverse and longitudinal cohorts of dogs [15,43]. The ultimate goal is to develop a blood-based biomarker panel that can be used easily in a clinical setting for the early detection and definitive diagnosis of the syndrome, as well as for the objective monitoring of disease progression and response to therapy.

Thirdly, more large-scale, prospective, randomised, placebo-controlled clinical trials are needed to rigorously evaluate the efficacy of both existing and novel therapeutic interventions [2,51]. These trials must account for the significant placebo effect often observed in studies relying on owner-reported outcomes by incorporating objective measures of cognition alongside behavioural rating scales [51]. Finally, further basic research is required to elucidate the more nuanced aspects of CCDS pathophysiology, particularly the precise role of neuroinflammation as a disease driver, and the molecular mechanisms that protect the canine brain from developing advanced tau pathology, as seen in human AD [9,10].

### 6.2. The Translational Value of CCDS Research

Studying CCDS is vital not only for improving the health of companion animals, but also for advancing human medicine. Although it has been shown that CCDS shares similarities with various neurodegenerative conditions in humans and should not be regarded as an exact counterpart of AD in dogs, it can offer clear advantages over other animal models [3,38,44].

As a spontaneous model of age-related neurodegeneration, the domestic dog offers a unique and powerful research platform that traditional laboratory animal models cannot provide [10,13]. As they share our homes, lifestyles and environmental exposures, dogs develop a form of dementia that more closely resembles the sporadic, late-onset form of AD—which accounts for over 95% of human cases—than any transgenic rodent model can [12,15].

This makes the canine model exceptionally well suited for testing novel preventive and therapeutic strategies. Findings from canine clinical trials investigating nutritional interventions, pharmacological agents such as senolytics, or novel approaches such as immunotherapy can provide crucial data on safety and efficacy in a large, genetically diverse mammal with naturally occurring disease [2,51]. Positive results from these trials could inform and accelerate the development of similar therapies for human AD, offering hope in the fight against one of the greatest public health challenges of our time [12,50].

### 6.3. Conclusions

Canine Cognitive Dysfunction Syndrome is a common and serious progressive medical condition that significantly compromises the welfare of ageing dogs. It is important for veterinary professionals and pet owners to recognise that the behavioural changes associated with CCDS are not an inevitable or ‘normal’ consequence of ageing, but rather the clinical manifestation of a treatable neurodegenerative disease. The key to managing this condition lies in taking a proactive and integrated approach to geriatric canine brain health.

This approach must be based on early detection, which can only be achieved through routinely implementing cognitive screening for all senior and geriatric canine patients. Once identified, a multimodal management plan should be implemented, combining the most appropriate evidence-based strategies from each therapeutic category: pharmacological intervention to address neurotransmitter and molecular deficits; targeted nutritional support to fuel the brain and combat oxidative stress; and a robust programme of environmental enrichment and behavioural management to promote neuroplasticity and reduce anxiety. Central to the success of this integrated approach is a strong partnership between veterinarian and client, based on clear communication and shared goals. By collaborating to diagnose CCDS early and manage it comprehensively, the veterinary profession can significantly improve quality of life and preserve the cherished human–animal bond for millions of ageing canine companions and their families [12].

## Figures and Tables

**Table 2:** Validated Owner-Based Questionnaires for the Assessment of CCD.

Feature	Canine Cognitive Dysfunction Rating (CCDR) Scale	CAnine DEmentia Scale (CADES)	References
Number of Items	13 items	17 items, plus 3 non-scored items	[45,46]
Key Domains Assessed	Assesses frequency and change in behaviours related to disorientation, social interactions, sleep–wake cycles, and house-soiling.	Assesses severity of signs across 4 domains: Spatial Orientation, Social Interactions, Sleep–Wake Cycles, and House Soiling.	[10,12,25,45,46]
Scoring System	Items scored on a 3-point or 5-point Likert scale. Total score is calculated with weighting for certain items, with a final adjusted score ranging up to 80.	Items scored on a 0–4 or 0–5 severity scale. Scores are summed within each domain and then a total score is calculated.	[25,45,46]
Cut-off Values	A total score of 50 or above is typically used as the threshold for a diagnosis of CCDS. Scores of 40–49 may indicate mild cognitive impairment or “at-risk” status.	Provides cut-off scores for different stages: Normal (0–7), Mild Cognitive Impairment (8–23), Moderate Cognitive Impairment (24–44), Severe Cognitive Impairment (45–80).	[4,45,46]
Key Strengths	Well validated and widely used in numerous clinical and research studies. Good diagnostic accuracy for identifying established CCDS.	Shown to be effective for separating different stages of CCDS, identifying earlier stages of impairment, and tracking disease progression over time.	[10,11,25,45]
Key Weaknesses	May be less sensitive for detecting very early or mild cognitive changes. Relies on owner subjectivity and recall	Relies on owner subjectivity and recall. May be more complex for owners to complete than the CCDR.	[12,45]

## Data Availability

No new data were created or analyzed in this study. Data sharing is not applicable to this article.

## References

[1] Dewey C.W., Davies E.S., Xie H., Wakshlag J.J. (2019). Canine Cognitive Dysfunction: Pathophysiology, Diagnosis, and Treatment. The Veterinary clinics of North America. Small Anim. Pract..

[2] Zakošek Pipan M., Prpar Mihevc S., Štrbenc M., Košak U., German Ilić I., Trontelj J., Žakelj S., Gobec S., Pavlin D., Majdič G. (2021). Treatment of canine cognitive dysfunction with novel butyrylcholinesterase inhibitor. Sci. Rep..

[3] Vitturini C., Cerquetella M., Spaterna A., Bazzano M., Marchegiani A. (2025). Diagnosis of Canine Cognitive Dysfunction Syndrome: A Narrative Review. Vet. Sci..

[4] Fast R., Schütt T., Toft N., Møller A., Berendt M. (2013). An observational study with long-term follow-up of canine cognitive dysfunction: Clinical characteristics, survival, and risk factors. J. Vet. Intern. Med..

[5] Landsberg G. (2005). Therapeutic agents for the treatment of cognitive dysfunction syndrome in senior dogs. Prog. Neuro-Psychopharmacol. Biol. Psychiatry.

[6] Salvin H.E., McGreevy P.D., Sachdev P.S., Valenzuela M.J. (2011). The canine cognitive dysfunction rating scale (CCDR): A data-driven and ecologically relevant assessment tool. Vet. J..

[7] Blanchard T., Eppe J., Mugnier A., Delfour F., Meynadier A. (2025). Enhancing cognitive functions in aged dogs and cats: A systematic review of enriched diets and nutraceuticals. GeroScience.

[8] Dewey C.W., Rishniw M., Brunke M.W., Gerardi J., Sakovitch K. (2024). Transcranial photobiomodulation therapy improves cognitive test scores in dogs with presumptive canine cognitive dysfunction: A case series of five dogs. Open Vet. J..

[9] Ozawa M., Chambers J.K., Uchida K., Nakayama H. (2016). The Relation between canine cognitive dysfunction and age-related brain lesions. J. Vet. Med. Sci..

[10] Vite C., Head E. (2014). Aging in the Canine and Feline Brain. Vet. Clin. N. Am. Small Anim. Pract..

[11] Bray E.E., Raichlen D.A., Forsyth K.K., Promislow D.E.L., Alexander G.E., MacLean E.L. (2023). Associations between physical activity and cognitive dysfunction in older companion dogs: Results from the Dog Aging Project. GeroScience.

[12] Pan Y., Kennedy A.D., Jönsson T.J., Milgram N.W. (2018). Cognitive enhancement in old dogs from dietary supplementation with a nutrient blend containing arginine, antioxidants, B vitamins and fish oil. Br. J. Nutr..

[13] Wrightson R., Albertini M., Pirrone F., McPeake K., Piotti P. (2023). The Relationship between Signs of Medical Conditions and Cognitive Decline in Senior Dogs. Animals.

[14] Pan Y. (2021). Nutrients, Cognitive Function, and Brain Aging: What We Have Learned from Dogs. Med. Sci..

[15] Milgram N.W., Zicker S.C., Head E., Muggenburg B.A., Murphey H., Ikeda-Douglas C.J., Cotman C.W. (2002). Dietary enrichment counteracts age-associated cognitive dysfunction in canines. Neurobiol. Aging.

[16] Cummings B.J., Head E., Afagh A.J., Milgram N.W., Cotman C.W. (1996). Beta-amyloid accumulation correlates with cognitive dysfunction in the aged canine. Neurobiol. Learn. Mem..

[17] Head E., Callahan H., Muggenburg B.A., Cotman C.W., Milgram N.W. (1998). Visual-discrimination learning ability and beta-amyloid accumulation in the dog. Neurobiol. Aging.

[18] Head E., McCleary R., Hahn F.F., Milgram N.W., Cotman C.W. (2000). Region-specific age at onset of beta-amyloid in dogs. Neurobiol. Aging.

[19] Pop V., Head E., Hill M.A., Gillen D., Berchtold N.C., Muggenburg B.A., Milgram N.W., Murphy M.P., Cotman C.W. (2010). Synergistic effects of long-term antioxidant diet and behavioral enrichment on beta-amyloid load and non-amyloidogenic processing in aged canines. J. Neurosci..

[20] Rofina J.E., van Ederen A.M., Toussaint M.J., Secrève M., van der Spek A., van der Meer I., Van Eerdenburg F.J., Gruys E. (2006). Cognitive disturbances in old dogs suffering from the canine counterpart of Alzheimer’s disease. Brain Res..

[21] Landsberg G.M., Nichol J., Araujo J.A. (2012). Cognitive dysfunction syndrome: A disease of canine and feline brain aging. Vet. Clin. N. Am. Small Anim. Pract..

[22] Iliff J.J., Wang M., Liao Y., Plogg B.A., Peng W., Gundersen G.A., Benveniste H., Vates G.E., Deane R., Goldman S.A. (2012). A paravascular pathway facilitates CSF flow through the brain parenchyma and the clearance of interstitial solutes, including amyloid β. Sci. Transl. Med..

[23] Tarasoff-Conway J.M., Carare R.O., Osorio R.S., Glodzik L., Butler T., Fieremans E., Axel L., Rusinek H., Nicholson C., Zlokovic B.V. (2015). Clearance systems in the brain-implications for Alzheimer disease. Nat. Rev. Neurol..

[24] Smith E.E., Greenberg S.M. (2009). Beta-amyloid, blood vessels, and brain function. Stroke.

[25] González-Martínez Á., Rosado B., Pesini P., Suárez M.L., Santamarina G., García-Belenguer S., Villegas A., Monleón I., Sarasa M. (2011). Plasma β-amyloid peptides in canine aging and cognitive dysfunction as a model of Alzheimer’s disease. Exp. Gerontol..

[26] Walsh D.M., Selkoe D.J. (2007). A beta oligomers—A decade of discovery. J. Neurochem..

[27] Shankar G.M., Li S., Mehta T.H., Garcia-Munoz A., Shepardson N.E., Smith I., Brett F.M., Farrell M.A., Rowan M.J., Lemere C.A. (2008). Amyloid-beta protein dimers isolated directly from Alzheimer’s brains impair synaptic plasticity and memory. Nat. Med..

[28] Panek W.K., Murdoch D.M., Gruen M.E., Mowat F.M., Marek R.D., Olby N.J. (2021). Plasma Amyloid Beta Concentrations in Aged and Cognitively Impaired Pet Dogs. Mol. Neurobiol..

[29] Haapasalo A., Kovacs D.M. (2011). The many substrates of presenilin/γ-secretase. J. Alzheimer’s Dis. JAD.

[30] Colle M.-A., Hauw J.-J., Crespeau F., Uchihara T., Akiyama H., Checler F., Pageat P., Duykaerts C. (2000). Vascular and parenchymal Abeta deposition in the aging dog: Correlation with behavior. Neurobiol. Aging.

[31] Schütt T., Toft N., Berendt M. (2015). Cognitive Function, Progression of Age-related Behavioral Changes, Biomarkers, and Survival in Dogs More Than 8 Years Old. J. Vet. Intern. Med..

[32] Head E. (2013). A canine model of human aging and Alzheimer’s disease. Biochim. Biophys. Acta.

[33] Smolek T., Madari A., Farbakova J., Kandrac O., Jadhav S., Cente M., Brezovakova V., Novak M., Zilka N. (2016). Tau hyperphosphorylation in synaptosomes and neuroinflammation are associated with canine cognitive impairment. J. Comp. Neurol..

[34] Siwak-Tapp C.T., Head E., Muggenburg B.A., Milgram N.W., Cotman C.W. (2007). Neurogenesis decreases with age in the canine hippocampus and correlates with cognitive function. Neurobiol. Learn. Mem..

[35] Ransohoff R.M., Brown M.A. (2012). Innate immunity in the central nervous system. J. Clin. Investig..

[36] Buccellato F.R., D’Anca M., Serpente M., Arighi A., Galimberti D. (2022). The Role of Glymphatic System in Alzheimer’s and Parkinson’s Disease Pathogenesis. Biomedicines.

[37] Campbell S., Trettien A., Kozan B. (2001). A noncomparative open-label study evaluating the effect of selegiline hydrochloride in a clinical setting. Vet. Ther. Res. Appl. Vet. Med..

[38] Marien M.R., Colpaert F.C., Rosenquist A.C. (2004). Noradrenergic mechanisms in neurodegenerative diseases: A theory. Brain Res. Rev..

[39] Pan Y., Larson B., Araujo J.A., Lau W., de Rivera C., Santana R., Gore A., Milgram N.W. (2010). Dietary supplementation with medium-chain TAG has long-lasting cognition-enhancing effects in aged dogs. Br. J. Nutr..

[40] Head E., Rofina J., Zicker S. (2008). Oxidative stress, aging, and central nervous system disease in the canine model of human brain aging. Vet. Clin. N. Am. Small Anim. Pract..

[41] Katina S., Farbakova J., Madari A., Novak M., Zilka N. (2016). Risk factors for canine cognitive dysfunction syndrome in Slovakia. Acta Vet. Scand..

[42] Dewey C.W., Rishniw M., Johnson P.J., Platt S., Robinson K., Sackman J., O’Donnell M. (2021). Canine cognitive dysfunction patients have reduced total hippocampal volume compared with aging control dogs: A comparative magnetic resonance imaging study. Open Vet. J..

[43] Yoon J.W., Nam C.S., Lee K.S., Dan T.J., Jeon H.J., Kang M.A., Park H.M. (2025). Evaluation of Blood-Based Diagnostic Biomarkers for Canine Cognitive Dysfunction Syndrome. Animals.

[44] Chapman B.L., Voith V.L. (1990). Behavioral problems in old dogs: 26 cases (1984–1987). J. Am. Vet. Med. Assoc..

[45] Ozawa M., Inoue M., Uchida K., Chambers J.K., Takeuch Y., Nakayama H. (2019). Physical signs of canine cognitive dysfunction. J. Vet. Med. Sci..

[46] Madari A., Farbakova J., Katina S., Zakošek Pipan T., Novak P., Weissova T., Novak M., Zilka N. (2015). Assessment of severity and progression of canine cognitive dysfunction syndrome using the CAnine DEmentia Scale (CADES). Appl. Anim. Behav. Sci..

[47] Dondi M., Bianchi E., Borghetti P., Di Lecce R., Gnudi G., Guarnieri C., Buffagni V., Ravanetti F., Saleri R., Corradi A. (2026). Canine Cognitive Dysfunction and Alzheimer’s Disease: Pathophysiological Relationships and the Impact of Glymphatic System Impairment on Neurodegeneration. Vet. Sci..

[48] Frampton M., Harvey R.J., Kirchner V. (2003). Propentofylline for dementia. Cochrane Database Syst. Rev..

[49] Siwak C.T., Gruet P., Woehrlé F., Muggenburg B.A., Murphey H.L., Milgram N.W. (2000). Comparison of the effects of adrafinil, propentofylline, and nicergoline on behavior in aged dogs. Am. J. Vet. Res..

[50] Alvarenga I., Panickar K., Hess H., McGrath S. (2023). Scientific Validation of Cannabidiol for Management of Dog and Cat Diseases. Annu. Rev. Anim. Biosci..

[51] Simon K.E., Russell K., Mondino A., Yang C.C., Case B.C., Anderson Z., Whitley C., Griffith E., Gruen M.E., Olby N.J. (2024). A randomized, controlled clinical trial demonstrates improved owner-assessed cognitive function in senior dogs receiving a senolytic and NAD+ precursor combination. Sci. Rep..

[52] Pan Y., Landsberg G., Mougeot I., Kelly S., Xu H., Bhatnagar S., Gardner Cari L., Milgram Norton W. (2018). Efficacy of a Therapeutic Diet on Dogs with Signs of Cognitive Dysfunction Syndrome (CDS): A Prospective Double Blinded Placebo Controlled Clinical Study. Front. Nutr..

[53] Benedetti R., Marchegiani A., Tambella A.M., Fruganti A., Serri E., Malfatti A., Spaterna A. (2019). Effects of chronic supplementation of homotaurine on cognitive processes and spatial cognition in aged dogs: Preliminary results. J. Vet. Behav..

[54] Manzano S., Agüera L., Aguilar M., Olazarán J. (2020). A Review on Tramiprosate (Homotaurine) in Alzheimer’s Disease and Other Neurocognitive Disorders. Front. Neurol..

